# “Suffering is only intolerable when nobody cares”… is it?

**DOI:** 10.1017/S1478951524001792

**Published:** 2025-01-13

**Authors:** Ana Cláudia Mesquita Garcia

**Affiliations:** Interdisciplinary Center for Studies in Palliative Care, School of Nursing, Federal University of Alfenas (UNIFAL-MG), Alfenas, Brazil; Interdisciplinary Cooperation for Ayahuasca Research and Outreach (ICARO), School of Medical Sciences, State University of Campinas (UNICAMP), Campinas, Brazil

“Suffering is only intolerable when nobody cares” (Saunders 2006). This statement, extensively reproduced in the field of Hospice and Palliative Care, is one of the most iconic phrases of Dame Cicely Saunders, considered the founder of the modern Hospice movement. The principles Saunders formulated for St. Christopher’s, the hospice she founded in the 1960s and 1970s for the care of people with terminal illnesses, are the foundation upon which the modern philosophy of Hospice and Palliative Care has developed (Clark 2018). The phrase carries a message of hope and support, suggesting that as long as a person is being cared for, their suffering can be tolerable. However, I have been questioning this statement for some time now. What I intend to expose in this text is precisely what leads me to question it, especially when it comes to the approach to existential suffering. I believe that the idea behind this phrase needs to be reconsidered because it seems to me that not all suffering becomes tolerable with care, no matter how attentive, qualified, and dedicated it may be. There are pains that reside in the deeper layers of the human soul, traumas rooted in the psyche, irreparable losses, and scars that time does not completely soften. In such situations, care, no matter how valuable and compassionate, may be insufficient to eliminate suffering entirely or even make it bearable. What leads me to this thought is the perception of the complexity of human suffering, evidenced by requests for euthanasia and assisted suicide (EAS) from people in developed countries, which theoretically have the best indices of quality of death and dying (Finkelstein et al. 2022), but still opt to end their lives.

Before continuing with this reasoning, dear reader, I would like to open a parenthesis. You may be wondering, especially if you are a professional in the field of Palliative Care: “who are you to question Cicely Saunders?” “Who are you in the bread line?” (Quem é você na fila do pão? – Brazilian Portuguese spelling) is a popular expression used in Brazil humorously and ironically to ask about someone’s importance or relevance in a given context. It is like asking “who are you to have this opinion?” or “what is your authority on this subject?” In this sense, recognizing my insignificance in the field, I use the words of Clarice Lispector – an important Brazilian writer – and I preemptively apologize for the “sin of thinking.” My intention in this text is not to criticize in the negative sense of the word, to issue a severe or unfavorable judgment on someone or something. But to reflect on the subject, after all, from Kuhn’s (1962) perspective on how the progress of science occurs, questioning paradigms is healthy and essential for this progress. I take this opportunity to thank you for the lifetime you are spending reading this text, which for me gives meaning to the lifetime I spent reflecting on the subject and translating the ideas into the words that give body to this manuscript. Now, I would like to continue the reasoning initiated earlier.

It is known that the spread of EAS decreases if adequate palliative care is organized, capable of greatly reducing or even eliminating chronic pain (Colombo and Dalla-Zuanna 2024). Observations of end-of-life practices in Switzerland, the Netherlands, and Belgium, for example, suggest that early interaction between doctor and patient, the renunciation of therapeutic obstinacy, and especially the use of pain therapy and, if necessary, deep sedation can delay the spread of EAS (Colombo and Dalla-Zuanna 2024). However, the majority of public opinion in the 21st century is still favorable to EAS practices, especially when it comes to an option to end suffering considered unsustainable (Colombo and Dalla-Zuanna 2024). Cases like those in Switzerland and the Netherlands show that even the general availability of the best palliative care is not capable of stopping the growth in demand for EAS (Colombo and Dalla-Zuanna 2024). This may possibly occur because, in the current state of knowledge, even the best palliative care available cannot prevent a significant minority of patients from dying without unbearable pain (Colombo and Dalla-Zuanna 2024; Zamora et al. 2019).

Thinking specifically about existential issues, these are more evident in end-of-life care, being a significant source of suffering for patients with serious illnesses, influencing the desire to hasten death or leading to suicidal ideation (Breitbart et al. 2004; Gaignard et al. 2023). In this sense, there is an ongoing debate about the advisability of reducing the level of consciousness and even euthanasia for the relief of existential suffering. According to a study aimed at clarifying the clinical practice of continuous deep sedation (CDS) for controlling psychoexistential suffering, dependence, loss of autonomy, and hopelessness were common reasons for the psychoexistential suffering that required CDS (Maeda et al. 2023). Doomen (2023) brings up the possibility of legalizing euthanasia for people experiencing existential suffering, discussing the issue of the value of life and whether the standard that life is normally something positive should be accepted. For Doomen (2023), the question is not whether there is a right to die but whether there is a duty (to continue) to live in the face of an unbearable suffering situation.

Yalom suggests that existential suffering, a conflict shared by all humanity, arises from the lack of resources to face the reality of human existence (Yalom 1980, 1988). I interpret Yalom’s statement about existential suffering as the suffering arising from the awareness of the limitations imposed by the condition of being human. This suffering is not limited to internal struggles or traumatic memories but emerges from the confrontation with the reality of existence. Yalom states that everyone is destined to experience not only the euphoria of life but also its darkness: disillusionment, aging, illness, isolation, loss, lack of meaning, painful choices, and death (Yalom 2022). Moving to a harsher and more pessimistic perspective, Romanian philosopher Emil Cioran, in his work “The trouble with being born,” expresses his skeptical view of the human condition (Cioran 1976). With his aphoristic style and profound reflections, Cioran offers a unique and disturbing perspective on existential dilemmas and the meaning of life. He argues that life has no intrinsic purpose or meaning and that suffering is an inevitable and central part of the human experience. He sees pain and anguish as inherent to the condition of being alive, making life a heavy burden. This idea is taken to the extreme by Cioran when he refers to birth as an act of imposing suffering, questioning the value of bringing new lives into the world knowing they will inevitably experience pain and despair.

In light of the above, I argue that care, more specifically the approach of palliative and compassionate care, can certainly bring comfort and relief in situations of suffering, making them essential tools to help those suffering from serious illnesses, especially those at the end of life. However, they will not always be able to completely eliminate (or make tolerable) the pain, understood here from the perspective of total pain, a concept also proposed by Saunders (Wood 2022). Obviously, whenever possible, suffering should be alleviated. But certainly, there will be situations where this will not be possible, whether due to the limitation of scientific knowledge, our limited understanding of the human soul, our limited understanding of the mystery of suffering (da Silva and Garcia 2024), or even due to choices made by the patient throughout their life that may culminate in suffering at the end of it. Regarding this last point, I sympathize with Elisabeth Kübler-Ross’s statement: “Dying is nothing to fear. It can be the most wonderful experience of your life. It all depends on how you have lived” (Elisabeth Kübler-Ross Foundation 2024). This statement removes the power to relieve suffering from the hands of health-care professionals and returns it to the patient, which, in my view, is something positive in recognizing the individual’s autonomy over their own life – which also implies the responsibility to bear the consequences of their choices – which can be something truly harsh. Of course, the role of the health-care professional is not to judge such choices but to support the patient, helping them in the best possible way to “carry their cross,” using here the biblical image of Simon of Cyrene – the one who helped Christ carry his cross to Calvary.

It is important to recognize that suffering is part of the human experience, and this can have its value (da Silva and Garcia 2024). Paul Ramsey (1970) states that the function of medicine is not to relieve the human condition of the human condition. In my view, overlooking this reality might reflect an overly simplistic or overly confident perspective. It takes humility to recognize the limits of science and, in certain cases, its impotence in the face of the complexity of the experience of being human and the mystery of suffering (da Silva and Garcia 2024).Recognizing these limits does not diminish the value of care but highlights the need for a more realistic and less romanticized approach to dealing with human suffering.

Instead of denying the persistence of certain pains, it is important to recognize and validate the suffering of others, even if it is not possible to eliminate it. Care is fundamental in mitigating suffering, but it does not always eliminate it or make it bearable. Suffering may persist, but certainly, the compassionate and welcoming presence of another human being can make this journey a little less arduous.
